# Clinical and epidemiological predictors of transmission in Severe Acute Respiratory Syndrome (SARS)

**DOI:** 10.1186/1471-2334-6-151

**Published:** 2006-10-18

**Authors:** Mark IC Chen, Angela LP Chow, Arul Earnest, Hoe Nam Leong, Yee Sin Leo

**Affiliations:** 1 Clinical Epidemiology, Communicable Disease Centre, Tan Tock Seng Hospital, Jalan Tan Tock Seng, Singapore; 2 Surveillance Branch, Communicable Diseases Division, Ministry of Health, College Road, Singapore; 3 Department of Internal Medicine, Singapore General Hospital, Outram Road, Singapore; 4 Department of Infectious Diseases, Communicable Disease Centre, Tan Tock Seng Hospital, Jalan Tan Tock Seng, Singapore

## Abstract

**Background:**

Only a minority of probable SARS cases caused transmission. We assess if any epidemiological or clinical factors in SARS index patients were associated with increased probability of transmission.

**Methods:**

We used epidemiological and clinical data on probable SARS patients admitted to Tan Tock Seng Hospital. Using a case-control approach, index patients who had probable SARS who subsequently transmitted the disease to at least one other patient were analysed as "cases" against patients with no transmission as "controls", using multivariate logistic regression analysis.

**Results:**

98 index patients were available for analysis (22 with transmission, 76 with no transmission). Covariates positively associated with transmission in univariate analysis at p < 0.05 included delay to isolation (Day 7 of illness or later), admission to a non-isolation facility, pre-existing chronic respiratory disease and immunosuppressive disease, need for oxygen, shortness of breath, vomiting, and higher lactate dehydrogenase levels and higher neutrophil counts. In the multivariate analysis, only three factors were significant: delay to isolation, admission to a non-isolation facility and higher lactate dehydrogenase levels of >650 IU/L (OR 6.4, 23.8 and 4.7 respectively).

**Conclusion:**

Clinical and epidemiological factors can help us to explain why transmission was observed in some instances but not in others.

## Background

Severe Acute Respiratory Syndrome (SARS) was the first emerging infectious disease of the new century with epidemic potential. First recognized on 26 Feb 2003, SARS spread rapidly and resulted in 8098 reported cases and 774 deaths in close to 30 countries [1]. While there was no endemic transmission in the majority of these countries, explosive outbreaks were observed in China, Hong Kong, Taiwan, Canada, Vietnam and Singapore. Ongoing research points to an existing animal reservoir for the virus [2,3], and future epidemics may hence sporadically emerge from this source [4].

A key feature in the epidemiology of SARS is the widespread variation in the number of secondary infections caused by each potentially infectious case. While multiple secondary infections were traced to single individuals in several super-spreading events [5-7], the majority of infected individuals did not cause any secondary infections [7].

A recent paper showed that the efficiency of outbreak control measures could be greatly enhanced if there were predictive methods for identifying infectious individuals [8]. However, a review by Yu and Sung noted that the key risk factors for transmission remain largely unknown [9]. While two previous papers attempted to identify risk factors for onward transmission from index patients [10,11], both studies were restricted to household contacts, and neither accounted for factors such as clinical presentation, immune status and disease severity in the index patient, all of which have been suspected to play a role in disease transmission [9].

In this study, we analysed epidemiological and clinical data on probable SARS patients admitted to Tan Tock Seng Hospital. We attempted to identify if any of these factors could explain if individual index patients had transmitted the disease by the time they were detected and isolated, and developed a simple model for explaining the variability in secondary transmission.

## Methods

We used data collected during the outbreak of SARS in Singapore from 1 March to 31 May 2003. Diagnosis of probable SARS was based on the standard WHO case definitions dated 1^st ^May 2003 [12]. Briefly, it required a patient to have fever, respiratory symptoms and a significant contact history, in the presence of a chest radiograph consistent with pneumonia or adult respiratory distress syndrome, or laboratory assays diagnostic of SARS. We defined an index patient as any individual who had been established, during contact tracing investigations, to have come into effective contact with susceptible individuals, be it in healthcare, household or other social settings. This study included all individuals who were part of the chain of transmission within Tan Tock Seng Hospital – i.e. all index patients causing secondary infections within (TTSH), as well as all individuals exposed in TTSH who, in the course of follow-up surveillance and contact tracing, were found to have contracted SARS, and hence also became index patients. Index patients within TTSH were identified from outbreak investigations by on-site epidemiologists [13]. The hospital contact tracing team was also updated on individuals exposed in TTSH who were subsequently confirmed to be infected. This information was obtained, either when these infected individuals returned to us for clinical assessment, or through the Ministry of Health in the event that these infected individuals presented elsewhere. The team then collated epidemiological information on dates of onset, admission and isolation for these index patients, and verified with other healthcare institutions and the Ministry of Health if these index patients had in turn caused any secondary transmission in either nosocomial or community settings.

Secondary infections were linked to an index patient if either of the following criteria were met:

- there was effective contact between the index patient and the secondarily infected patient, either as recalled by the index patient or the secondarily infected patient, or documented in medical notes (eg. of physical examination of an index patient by a healthcare worker who became secondarily infected)

- there were movement records indicating that effective contact could have occurred between an index patient and a secondarily infected patient, with the same movement records indicating that no other index patients could be an alternative source of infection Eg. an index patient sharing the same room/cubicle as a secondarily infected patient, even if neither patient could give a history of direct contact with each other, as long as no other infectious SARS cases were detected in the same general area

In situations where a secondary infection could be linked to more than one index case, the first index patient with whom the secondary infection came into effective contact with was considered to be the source of the infection. Effective contact was defined as conversation or physical contact within a 1 metre distance, where the index patient had to already be symptomatic from SARS, and the onset of symptoms for the secondarily infected patient had to be 2 to 10 days after the exposure which presumptively led to the infection.

The above criteria were then used to classify all index patients as to whether the index patient had transmitted SARS to one or more other individuals, which was our outcome of interest. In the case-control analysis, index patients who caused at least one secondary infection were analysed as "cases"; all index patients who did not cause any secondary infections were analysed as "controls".

**Figure 1:**
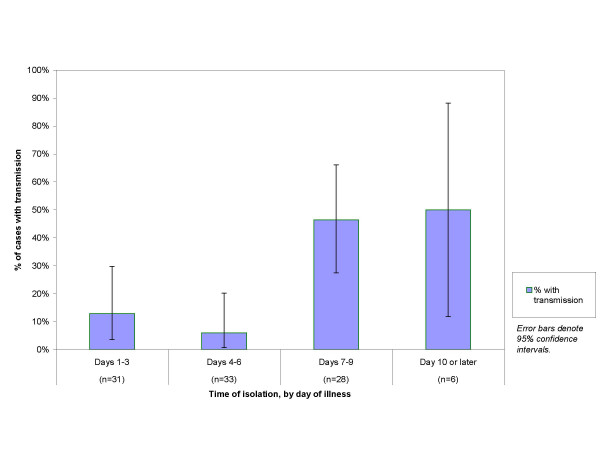
Proportion of cases with secondary transmission, by time of isolation.

Exposure variables of interest included the demographic, epidemiological and clinical features associated with individual index patients. Epidemiological variables were the context of the exposure to SARS for that index patient, the date of illness onset relative to the implementation of personal protective equipment (PPE) across the entire hospital on 22^nd ^Mar 2003, delayed isolation, and admission to a non-isolation ward. We defined delayed isolation as admission to isolation wards on Day 7 of illness or later, as an exploratory analysis of the data showed a sharp rise in the proportion causing secondary infections from Day 7 (Figure 1). Isolation wards were facilities where visitors were disallowed, and where healthcare staff wore specialized personal protective equipment while managing patients. All other wards are hereafter referred to as non-isolation wards. This included general wards and intensive care units (ICU) and high dependency (HD) wards where isolation precautions had not been instituted by the time the index patient for that ward was admitted.

Clinical signs and symptoms, as well as laboratory and radiological investigations, were obtained from a clinical database maintained for all probable SARS patients admitted to TTSH. Index patients were coded on whether key clinical signs and symptoms were absent or present on the date the patient was moved to isolation. For laboratory investigations, we used the results of tests carried out closest in time to the date of isolation. Co-morbid chronic illnesses were grouped as those with possible impact on respiratory function (pre-existing ischemic heart disease/congestive cardiac failure, chronic obstructive pulmonary disease), and those that could suppress the immune status (diabetes mellitus, chronic renal failure, malignancies, chronic immunosuppressive therapies).

We then identified factors significantly associated with secondary transmission through a univariate analysis. In the multivariate analyses, we started from the most significant factor identified in the univariate analysis, and then performed a forward step-wise regression, using the likelihood ratio test to see if inclusion of the next covariate significantly improved the fit of the multivariate model. Odds ratio and their 95% confidence intervals are presented as estimates of the effect sizes. The final multivariate model was then used to model the probability of transmission for combinations of individual patient characteristics.

All statistical analyses were performed using Stata V9.0 (Stata Corp, Tex) for Windows, with the level of significance set at 5%.

### Ethical Approval

Ethical approval for the clinical database and its subsequent use in this study was granted by the TTSH Hospital SARS Clinical Management Workgroup and the institutional representative for the hospital.

## Results

In all, 106 individuals satisfied our study inclusion criteria of being part of the chain of transmission within TTSH. Epidemiological investigations identified secondary transmission in 24 index patients, including 4 which were involved in super-spreading events; 10 of the 24 index patients caused secondary transmission within TTSH itself, while another 14 index patients caused secondary transmission in other healthcare institutions and/or the community. Two index patients with secondary transmission, and six index patients without secondary transmission, had to be excluded due to missing information on key variables. In all, 22 index patients who transmitted the disease were analysed as "cases", against 76 index patients with no identifiable secondary transmission serving as the "controls".

Table 1 presents the characteristics of our study population. In all, 58% were infected in the course of their duty as healthcare workers. Another 10% were infected while admitted as inpatients of TTSH for unrelated medical conditions and, with the exception of the imported index patient, the remainder was infected while visiting friends and relatives in TTSH. 90% of our cases eventually developed radiographic abnormalities, and positive confirmatory tests for SARS-CoV infection were available for 93% of the study patients. Median values for the laboratory investigations fell within the accepted reference ranges; for all subsequent analysis, we compared index patients with the most extreme quartile of results against all other index patients.

**Table 1:** Demographic, epidemiological, clinical, and laboratory features of study patients

**Demographics**	**n**	**(%)**
Age		
- < = 24	23	23.5%
- 25 to 49	61	62.2%
- 50 and above	14	14.3%
		
Female gender	79	80.6%
		
Non-Chinese ethnicity	40	40.8%
		
**Epidemiological features**	**n**	**(%)**
Context of exposure to SARS		
- healthcare worker	57	58.2%
- hospital inpatient	10	10.2%
- *others	31	31.6%
		
Date of onset		
- 21/3/03 or earlier (before universal PPE)	78	79.6%
- 22/3//03 or later (after universal PPE)	20	20.4%
		
Delayed isolation (on Day 7 or later)	34	34.7%
		
Ever admitted to a non-isolation ward	12	12.2%
		
Ever admitted to a non-isolation ward	12	12.2%
		
**Clinical features**	**n**	**(%)**
Chronic illness		
- chronic respiratory illness	7	7.1%
- disease with immunosuppression	10	10.2%
Disease severity at time of isolation		
- had an abnormal chest radiograph	78	79.6%
- required oxygen therapy	14	14.3%
- admitted to intensive care or high dependency	4	4.1%
- intubated	2	2.0%
Signs and symptoms at time of isolation		
- temperature > = 38°C	74	75.5%
- fever	95	96.9%
- cough	55	56.1%
- dyspnoea	24	24.5%
- vomiting	17	17.3%
- diarrhoea	14	14.3%
		
**Chest radiograph and confirmatory tests for SARS**		
- ever had abnormal CXR	88	89.8%
- positive PCR specimen	13	13.3%
- positive serology	91	92.9%
		
**^Other laboratory investigations**	**Median**	**(IQR)**
- highest alanine aminotransferase (7–36 IU/L)	21	(14–41)
- highest lactate dehydrogenase (200–500 IU/L)	447	(348–650)
- lowest platelet count (160-390*10^9/L)	184	(145–220)
- highest neutrophil count (4-10*10^9/L)	3.36	(2.35–5.04)
- lowest lymphocyte count (1.5-4.3*10^9/L)	0.93	(0.66–1.21)

*others: original imported index patient and hospital visitors

^reference ranges given in brackets

Table 2 compares index patients with and without transmission. None of the demographic characteristics were significantly associated with increased risk of transmission. However, index patients originally infected as inpatients were more likely to transmit the disease. Delay to isolation and admission to a non-isolation facility were both strongly associated with transmission. In terms of clinical characteristics, pre-existing chronic respiratory disease and immunosuppressive disease were more likely to be associated with increased risk of transmission. There were also indications that index patients with more severe illness were more likely to transmit the disease; while 4/22 (18%) of the index patients who transmitted the disease had been admitted to ICU or HD by the time of isolation, this was observed in none of the index patients who did not transmit the disease (p = 0.002, Fisher's exact test). Moreover, the need for oxygen, shortness of breath, vomiting, and higher lactate dehydrogenase levels and higher neutrophil counts, were all more frequent in index patients who transmitted the disease.

**Table 2:** Univariate analysis of factors associated with transmission of SARS

	**Patients with secondary transmission**		**Patients with no secondary transmission**				
	**(n = 22)**		**(n = 76)**				

**Factors**	**n**	**%**	**n**	**%**	**OR**	**95%CI**	**p-value**

**Demographics**							
Age							
- < = 24	4	18.2%	19	25.0%	1.00	(Ref)	
- 25 to 49	12	54.5%	49	64.5%	1.16	(0.33–4.06)	0.812
- 50 and above	6	27.3%	8	10.5%	3.56	(0.79–16.14)	0.099
							
Female gender (vs male)	19	86.4%	60	78.9%	1.69	(0.44–6.43)	0.442
							
Non-Chinese ethnicity (vs Chinese)	6	27.3%	34	44.7%	0.46	(0.16–1.31)	0.148
							
**Epidemiological features**							
							
Context of exposure to SARS							
- healthcare worker	7	31.8%	50	65.8%	1.00	(Ref)	
- hospital inpatient	6	27.3%	4	5.3%	10.70	(2.41–47.6)	0.002
- *others	9	40.9%	22	28.9%	2.92	(0.97–8.85)	0.058
							
Date of onset							
- 21/3/03 or earlier (before universal PPE)	18	81.8%	60	78.9%	1.00	(Ref)	
- 22/3//03 or later (after universal PPE)	4	18.2%	16	21.1%	1.61	(0.49–5.32)	0.437
							
Delayed isolation (on Day 7 or later)	16	72.7%	18	23.7%	8.59	(2.93–25.23)	<0.001
							
Ever admitted to a non-isolation ward	11	50.0%	1	1.3%	75.00	(8.8–639.18)	<0.001
							
**Clinical features**							
							
Chronic illness							
- chronic respiratory illness	5	22.7%	2	2.6%	10.88	(1.94–60.92)	0.007
- disease with immunosuppression	5	22.7%	5	6.6%	4.18	(1.09–16.08)	0.038
							
Disease severity at time of isolation							
- had an abnormal chest radiograph	15	68.2%	63	82.9%	0.44	(0.15–1.3)	0.138
- required oxygen therapy	7	31.8%	7	9.2%	4.60	(1.4–15.08)	0.012
- admitted to ICU or HD	4	18.2%	0	0.0%	NC		
- intubated	2	9.1%	0	0.0%	NC		
							
Signs and symptoms at time of isolation							
- temperature > = 38oC	16	72.7%	58	76.3%	0.83	(0.28–2.43)	0.731
- fever	21	95.5%	74	97.4%	0.57	(0.05–6.57)	0.65
- cough	13	59.1%	42	55.3%	1.17	(0.45–3.06)	0.75
- dyspnoea	9	40.9%	15	19.7%	2.82	(1.01–7.81)	0.047
- vomiting	8	36.4%	9	11.8%	4.25	(1.4–12.95)	0.011
- diarrhoea	6	27.3%	8	10.5%	3.19	(0.97–10.48)	0.056
							
**^Laboratory investigations**							
							
- alanine aminotransferase >41IU/L	7	31.8%	17	22.4%	1.62	(0.57–4.61)	0.367
- maximum lactate dehydrogenase >650IU/L	14	63.6%	10	13.2%	11.55	(3.87–34.49)	<0.001
- minimum platelet count < = 184*10^9/L	8	36.4%	16	21.1%	2.14	(0.77–6)	0.147
- maximum neutrophil count >5.04*10^9/L	11	50.0%	13	17.1%	4.85	(1.74–13.53)	0.003
- minimum lymphocyte count < = 0.93*10^9/L	9	40.9%	15	19.7%	2.82	(1.01–7.81)	0.047

*others: original imported index patient and hospital visitors

^unit of measurement as in Table 1

The results of the multivariate logistic regression are presented in Table 3. On multivariate analysis, the strongest predictor of transmission was admission to a non-isolation ward, followed by delay in isolation, and having a lactate dehydrogenase value >650IU/L. No significant interactions were identified between these three covariates.

**Table 3:** Multivariate analysis of factors associated with transmission

**Features**	**OR (95% CI)**	**p-values**
**Epidemiological features**		
		
Delayed isolation (on Day 7 or later)	6.43 (1.66–24.84)	0.007
		
Ever admitted to a non-isolation ward	23.83 (2.41–235.27)	0.007
		
**Laboratory investigations**		
- maximum lactate dehydrogenase >650IU/L	4.73 (1.14–19.65)	0.032

We used the coefficients given in Table 3 to construct a tool for predicting if an index patient would have caused transmission by the time he/she is identified and isolated. The predicted probabilities *(p*_*i*_) of transmission in Table 4 are calculated for the i^th ^individual (i = 1 to 98), using the equation below,

**Table 4:** Predictive model showing probability of transmission for index patients with different combinations of risk factors

**Risk factor combination, sorted by decreasing probability of transmission**	**Total index patients in group**	**Cumulative % of all patients**	**Predicted probability of transmission**	**Expected no. with transmission**	**Observed no. with transmission**
Delayed isolation + Admitted non-isolation + lactate dehydrogenase >650 IU/L	7	7.1%	0.969	6.78	6
Delayed isolation + Admitted non-isolation	2	9.2%	0.867	1.73	2
Admitted non-isolation + lactate dehydrogenase >650 IU/L	3	12.2%	0.828	2.48	3
Delayed isolation + lactate dehydrogenase >650 IU/L	6	18.4%	0.565	3.39	3
Admitted non-isolation only	Nil	18.4%	0.504	-	-
Delayed isolation only	19	37.8%	0.215	4.09	5
Lactate dehydrogenase >650 IU/L only	8	45.9%	0.168	1.34	2
No risk factors	53	100.0%	0.041	2.17	1

ln⁡(pi1−pi)=−3.15+1.86x1i+3.17x2i+1.55x3i
 MathType@MTEF@5@5@+=feaafiart1ev1aaatCvAUfKttLearuWrP9MDH5MBPbIqV92AaeXatLxBI9gBaebbnrfifHhDYfgasaacH8akY=wiFfYdH8Gipec8Eeeu0xXdbba9frFj0=OqFfea0dXdd9vqai=hGuQ8kuc9pgc9s8qqaq=dirpe0xb9q8qiLsFr0=vr0=vr0dc8meaabaqaciaacaGaaeqabaqabeGadaaakeaacyGGSbaBcqGGUbGBdaqadaqaamaalaaabaGaemiCaa3aaSbaaSqaaiabdMgaPbqabaaakeaacqaIXaqmcqGHsislcqWGWbaCdaWgaaWcbaGaemyAaKgabeaaaaaakiaawIcacaGLPaaacqGH9aqpcqGHsislcqaIZaWmcqGGUaGlcqaIXaqmcqaI1aqncqGHRaWkcqaIXaqmcqGGUaGlcqaI4aaocqaI2aGncqWG4baEcqaIXaqmdaWgaaWcbaGaemyAaKgabeaakiabgUcaRiabiodaZiabc6caUiabigdaXiabiEda3iabdIha4jabikdaYmaaBaaaleaacqWGPbqAaeqaaOGaey4kaSIaeGymaeJaeiOla4IaeGynauJaeGynauJaemiEaGNaeG4mamZaaSbaaSqaaiabdMgaPbqabaaaaa@5873@

where *x*1, *x*2, and *x*3 are indicator variables taking on the values of 1 for those with delayed isolation, admission to a non-isolation ward, and lactate dehydrogenase > 650 IU/L respectively; the term takes the value of 0 in the absence of each respective indicator.

The results of the predictive model (Table 4) illustrate the wide variability in transmission observed between index patients. While index patients with two or more risk factors have a predicted probability of transmission exceeding 0.5, they comprise only 20% of our study population. If we use the model in a predictive fashion, we see that, under the best-case scenario, when no risk factors are present at the point of isolation, the predicted probability that transmission has occurred is less than 5%. In contrast, in a situation where all the three significant risk factors are present, the model predicts that there is a 97% chance of secondary transmission having occurred.

An ROC for the model was plotted (not shown). The area under the curve was 0.903 (95%CI 0.832–0.974), indicating that the model had a very good ability to discriminate index patients with secondary transmission from those with no secondary transmission.

## Discussion

Our study confirms previous observations that the majority of SARS transmission is caused by a minority of cases [7]. Moreover, we have identified several key factors associated with an increased probability of transmission, and show that the main determinants were delayed isolation (Day 7 or later), admission to a non-isolation ward, and higher lactate dehydrogenase values at the time of isolation. These findings are important in several ways.

Firstly, our findings improve the current understanding of SARS transmission. While the importance of delayed isolation has been previously noted by Lipsitch and colleagues [14], we note that the strongest predictor for transmission having occurred is admission to a non-isolation ward. This has previously been suspected based on outbreak descriptions from healthcare settings. For example, in an outbreak of SARS among hospital workers in a community hospital in Hong Kong, it was observed that most staff was infected from exposure to patients with unsuspected SARS in non-isolation wards [15]. Similar observations of nosocomial transmission arising from unsuspected, non-isolated cases of SARS have been made across different countries and different hospital settings. These include other community hospitals [16,17], acute care wards [5,18,19], intensive care units [20,21], and emergency rooms [22,23]. While such descriptive accounts may be faulted on the basis of reporting biases, we were able to confirm through this analytic study that index patients admitted without isolation precautions were much more likely to result in secondary transmission of SARS.

In addition, we are able to show the association of patient factors with transmission probability. While multiple symptoms and clinical manifestations are significantly associated with transmission in the univariate analysis, variables such as oxygen dependency, prior history of co-morbid illness, higher neutrophil counts and lactate dehydrogenase levels are correlated to each other and to disease severity [24,25]. It was not surprising therefore that, in the multivariate analysis, only the strongest predictor of disease severity is retained. Higher levels of lactate dehydrogenase had previously been found to be one of the best correlates of severe disease [26]. As higher viral loads have also been found to be associated with more severe disease [27], it would hence be reasonable to hypothesize that higher viral loads may be the underlying mechanism for the increased risk of transmission in patients who are more severely ill.

Although no one has directly linked viral load to infectiousness, others have previously observed an association between disease severity in the index patient and disease transmission. Shen and colleagues found an association between disease severity, defined as eventual progression to death, and the extent of transmission, although their findings were based only on a univariate analysis of four super-spreading events against 73 other case-patients [5]. Numerous outbreak reports, including incidents of transmission in intensive care units [19,21], the key super-spreading event in hotel M [28], the outbreak in Vietnam [29], as well as the flight from Hong Kong to Beijing on which 13 passengers were infected [30], all involved index patients who succumbed to their illness shortly after.

It would appear therefore that, not only is disease severity associated with the probability of transmission, but it is also a factor in the intensity of transmission. The link may lie in the number of susceptible contacts exposed to such severely ill patients, and follow-up studies on the Singapore data are in progress to verify this hypothesis.

Our findings have several key implications. Firstly, because admission to non-isolation wards was the most important predictor of secondary transmission, good case-detection and infection control at the point of entry into the inpatient healthcare setting is a priority area in the management of SARS outbreaks. Secondly, we have shown through our predictive model that there is a vast and real difference in the risk of transmission between different index patients. In the majority of cases, with no risk factors, the predicted probability of secondary transmission is small, whereas in those with a combination of the various factors, the risk of transmission is many times greater. Moreover, as Lloyd-Smith and colleagues pointed out, this variability in transmission risk amongst cases is not restricted to SARS, but may be common to other infectious diseases [8]. This raises the possibility that management of future outbreaks of emerging and re-emerging infectious diseases should include real-time analysis of clinical and epidemiological correlates similar to the one we have performed, so as to facilitate targeted outbreak interventions.

We acknowledge several limitations and weaknesses in our study. Firstly, the assessment of epidemiological linkages was partially subjective, and dependant on the information volunteered by index patients and family members. However, close to 7 out of every 10 infected contacts were healthcare workers and household members (data not shown), for which epidemiological linkages were obvious, and we are hence confident that the 22 index patients analysed as "cases" did transmit SARS to at least one other individual. The key weakness of our study therefore, is whether some of the index patients analysed as "controls" actually caused undetected transmission. With regards to this, we note that, within TTSH itself, 18 of 105 individuals infected in TTSH could not be definitely linked to a single index patient by the criteria we used. Of these, 3 were suspected to be linked to 3 of the index patients which were already classified as "cases" on the basis of having infected other individuals, 10 were exposed to multiple index cases within one single ward where a super-spreading event had occurred, and in 5 individuals we could not trace the infection to a specific source case or even a definite location within TTSH. In addition, any of our 76 controls could also have caused asymptomatic secondary infections, and we hence acknowledge that some bias from misclassification of "cases" as "controls" would be inherent in our study. However, we believe that comparing the index patients which caused secondary transmission against a set of imperfect controls is a superior approach to simply drawing qualitative conclusions on possible factors associated with transmission based on descriptions of index patients with transmission, and we have done so while attempting to minimise biases by the use of fairly stringent criteria in linking any index patients and secondary infections. We also acknowledge that recall and interviewer bias could have affected ascertainment of symptomatology and the timing onset. However, the other key findings of admission to a non-isolation ward and having higher lactate dehydrogenase levels are fairly objective criteria. Thirdly, the number of patients who transmitted was small. Consequently, the study may have lacked power to identify some important risk factors. The small number of events also contributed to the wide confidence intervals for some of the odds ratios. Also, unmeasured confounders could have affected the results of the study. In particular, other than including a variable for admission to non-isolation wards, we did not measure the characteristics of exposed susceptible individuals and the environmental circumstances surrounding the index patients. These would have included the size and configuration of households exposed to these index patients, and for patients admitted to non-isolation wards, the type of wards and any procedures the index patients were subjected to, as these may have resulted in differences in opportunities for transmission. Household factors have been explored in other work [10,11] but hospital-related factors remain a subject for future research, and would require a larger dataset of index patients admitted to non-isolation wards, or methodologies treating entire wards rather than individual index patients as the unit of analysis. In the absence of more detailed data and a larger dataset on household and hospital-related circumstances surrounding the index patients, we have opted simply to group the combined effect of transmission in these settings, and hence cannot rule out the possibility that unmeasured factors in the household and hospital environment may have confounded our findings. However, while we could not adjust for these potential confounders, it must be noted that those factors found to be significant in the multivariate analysis had reasonably high odds ratios, indicating that any residual confounding are less likely to affect our conclusions. Finally, we acknowledge that the predictive model derived from the study needs to be validated externally against retrospective data from other countries, or through prospective data, in the unfortunate event that SARS re-emerges. This is particularly since the infectiousness of the virus is likely to vary between strains. Inter-strain variations in infectiousness was not likely to be an important confounder in our study, since all the index cases here were within a few generations of the original imported case which started the outbreak in Singapore. However, any re-emergence of the virus would possibly involve a strain with fairly different features, and hence extreme caution should be exercised in generalising our results to any future outbreaks. However, the final model presented in this study does explain to a large extent the variability in transmission that was observed during the outbreak, and the prominent role of healthcare institutions and severely ill patients in the propagation of the outbreak.

In the event of a future outbreak of SARS, our analysis provides a starting point for risk assessment and risk communication. Our study also makes the case for the timely collation and correlation of clinical with epidemiologic data. In any future outbreak of emerging and re-emerging infectious diseases, such analyses may well allow index patients with higher transmission probability to be identified, and consequently facilitate better targeting of outbreak control measures.

## Conclusion

In this observational study amongst probable SARS patients, we have found that delayed isolation, admission to a non-isolation ward, and higher lactate dehydrogenase levels were independently and significantly associated with increased transmission risk. Our paper is the first analytic study to demonstrate the above associations while adjusting for possible confounders. Our final regression model based on these three factors, while not perfect, does explain some of the variability in transmission between cases. It also hints that a similar approach may be possible for other emerging and re-emerging infectious diseases, if data on epidemiological data on transmission can be linked to clinical and laboratory data in real-time.

## Competing interests

All the author(s) do not have any financial or non-financial competing interests.

## Authors' contributions

MIC conceived and designed the study. AE was involved in the statistical analysis. All authors contributed to the interpretation of the results and the writing of the manuscript.

## Pre-publication history

The pre-publication history for this paper can be accessed here:


